# Colostrum avoidance practice among primipara mothers in urban Northwest Ethiopia. A cross-sectional study

**DOI:** 10.1186/s12884-021-03623-w

**Published:** 2021-02-11

**Authors:** Tilksew Ayalew, Eden Asmare

**Affiliations:** 1 Department of Pediatrics and Child Health Nursing, College of Medicine and Health Sciences, Bahir Dar University, Bahir Dar, Ethiopia; 2 Department of Midwifery, College of Medicine and Health Sciences, Bahir Dar University, Bahir Dar, Ethiopia

**Keywords:** Breastfeeding, Colostrum avoidance, Primipara, Bahir Dar, Ethiopia

## Abstract

**Background:**

Breast milk is the ideal and complete form of nutrition for infants colostrum contains all the necessary nutrients for infants’ growth and development and antibodies that can protect from many childhood illnesses. Understanding the extent of and barriers to colostrum avoidance in Ethiopia is important for learning how to best improve optimal breastfeeding. No single study has been conducted on primigavida mothers in the country. Therefore, this study aimed to assess the rate of colostrum avoidance practice and associated factors among primigavida mothers.

**Method:**

A community- based cross-sectional study was conducted from March to April 2016 among (*n* = 398) randomly selected primigavida mothers in Bahir Dar city, northwest Ethiopia. Data were collected using a structured interviewer-administered questionnaire and analyzed using SPSS version25. Bivariate and multivariate logistic regression analyses were carried out. Odds ratio with 95% confidence interval was used to measure the strength of association. Statistical significance was declared at *P*-value ≤0.05.

**Results:**

Out of 398 primipara mothers, 8.8% discarded colostrum. The most common reasons for discarding colostrum were; yellow and creamy (39.2%), bad for infant (35.2%), traditional/cultural reason (17.1%) and infant unable to feed (8.5%). Married mothers (AOR = 4.52, 95%CI: 1.13, 18.16), unemployed mothers (AOR = 3.46, 95%CI: 1.15, 10.51), mothers underwent normal delivery (AOR = 5.20, 95%CI: 1.87, 20.90) and mothers who initiated breastfeeding within 1 h (AOR = 2.79, 95%CI: 0.96, 8.16) were less likely to discard colostrum.

**Conclusion:**

The current study revealed that colostrum was discarded by 8.8% of primipara mothers. Primipara mothers who were married, unemployed, underwent normal delivery and initiated breastfeeding within 1 h were less likely to discard colostrum. These results suggest that multi-sectorial and multi-disciplinary approaches are needed to decrease colostrum avoidance among primipara mothers in Ethiopia.

## Background

Breast milk is the ideal and complete form of nutrition for infants. World Health Organization (WHO) and United Nation Fund for Children (UNICEF)) recommended early initiation of breast feeding, and exclusive breast feeding for 6 months, and feeding breast milk for 2 years and beyond with appropriate and adequate complementary foods [1, 2].

Breast feeding of new born infants has important implications for immediate and future health especially, in developing nations like Ethiopia where there is high rate of malnutrition, infectious disease and mortality among children [3]. Sub optimal breast feeding practices including colostrum avoidance significantly impair the health, development and survival of children less than 5 years of age [2]. A systematic review and meta-analysis conducted on the effect of optimal breast feeding on child mortality showed that infants who were not exclusively breastfed and given continued breastfeeding had significantly higher risk of all-cause and infection mortality compared to their counter parts [4]. Another evidence from the lancet series on breast feeding revealed that universal breast feeding can prevent the deaths of 823,000 children and 20,000 mothers each year along with $US300 billion economic saving [5].

Colostrum is the first and most immunologically protective secretion of the mammary glands, which is highly nutritious, easily digestible, and act as a natural vaccine various health treats in infants [3, 6]. Evidence from all over the world revealed that neonatal and postnatal deaths were found to be decreased among infants who fed colostrum [7]. However, colostrum avoidance which includes delayed initiation of breast-feeding; pumping and discarding colostrum, and/or wet nursing [3, 6] has been reported across the globe various places such as Indonesia [8], Egypt [6], India [9, 10], Ethiopia [3, 11–16].

Mothers across the world particularly in the developing countries including Ethiopia discard colostrum due to different reasons. Some of the reasons of discarding colostrum are; colostrum is unclean, heavy and hard for digestion, causes abdominal crap and diarrhea, bad lack for family, the traditional belief that colostrum should not be fed until the placenta is passed, viewing it as it is a puse collected in the breast during pregnancy, seeing it having no nutritional value. Some women do not have specific reasons to discard colostrum other than traditional practice [3, 6, 7, 9].

Colostrum avoidance is the common nutritional malpractice in Ethiopia [3]. Studies in different regions of the country revealed that the rate of colostrum avoidance among Ethiopian mothers is (6–76.9%) [3, 11, 16–18].

Evidences showed that primipara mothers; especially teenage primipara mothers are more likely to practice suboptimal breastfeeding than multipara mothers. A study conducted in Indonesia among primigavida mothers revealed 7 % of the participants discarded colostrum and most participants (56%) did not have sufficient information about optimal breastfeeding [8]. Another study conducted in Pakistan has reported more than two-third (67%) of primigavida mothers had discarded colostrum and offered prelacteal foods to their new born babies [19]. Gebremeskel et al. have shown in their work that primipara mothers are more likely to practice prelacteal feeing than multipara mothers. They have also revealed that mothers who discarded colostrum were more likely to practice prelacteal feeding compared to mothers who fed colostrum to their new born babies [20].

In Ethiopia, even though few studies are conducted to investigate rate of colostrum avoidance and its associated factors, no researcher tried to investigate the situation in the population primipara mothers. Therefore; the objective of this study was to determine the rate of colostrum avoidance and associated factors among primigavida mothers in northwest, urban Ethiopia.

## Methods

### Study area and setting

This study was conducted in Bahir Dar city administration from March to April, 2016. Bahir Dar city is located 578 km northwest of Addis Ababa, the capital city of Ethiopia. According to the 2007 Census conducted by the Central Statistical Agency of Ethiopia, Bahir Dar city has a total population of 221,991, of whom 49% are men and 51% are women. From female population, around 66% were reproductive age groups. During the study period, a total of 2500 primi-gavida mothers were living in in Bahir Dar City. The city has nine administrative sub cities. It has one public specialized referral hospital, one public general hospital, two private hospitals, ten health centers which give services for the population of the city [21].

### Study design and population

A community-based, cross-sectional study design was used to determine the rate of colostrum avoidance among primigavida mothers living in Bahir Dar city, northwest Ethiopia. Randomly selected primigavida mothers between the age of 15 and 49 years of age, with infants younger than 6 months of age, and who live at list for 6 months in Bahir Dar city were included. Primigavida mothers who lived less than 6 months in Bahir Dar city, who are critically ill or unable to communicate, who were under 16 years old without parents or guardians were excluded.

### Sample size and sampling procedure

A total sample of 400 mothers was calculated using Yamane’s formula (*n* = N/1 + N (e^2^) by considering the following assumptions; *P* = 0.5, a 95% level of confidence, *N* = 2500(population size) and e = ±5%(level of precision) [22]. First, a total of 2500 mother-infant dyads of primi-gavida mothers were accessed and listed from Health Information System (HIS) of Bahir Dar city health bureau in collaboration with the local health extension workers of the city administration. Then, mothers were sorted and listed in their respective sub-city. Besides, the total sample size (*n* = 400) was proportionally allocated to size to each sub-city. Finally, the study participants were selected by using simple random sampling method from each sub-city. Data on infants were gained from infants’ mothers and by reviewing birth certificate of infants.

### Data collection and data quality assurance

Data were collected using a pre-tested, structured, and interviewer-administered questionnaire which was adopted from previous studies [23–25]. Mothers were interviewed at their households. The English version of the questionnaire was prepared first. Then, language experts translated it to the local language (*Amharic*) and back to the English to check consistency and accuracy. Three diploma nurses and two Bachelor of Science nurses were recruited as data collectors and supervisors respectively. To ensure data quality, training was given for data collectors and supervisors for two consecutive days on the overall content of the questionnaire, how to approach participants, and the data collection process. Assigned supervisors closely managed the data collection process. A pre-test was done on 10% of the calculated sample size of women out of study area and readjustment was done on the questionnaire.

### Measurement

#### Variables

The dependent variable in this study was colostrum avoidance and the independent variables were socio-demographic characteristics, maternal health care service utilization, breastfeeding-related factors, and other factors.

#### Operational definitions

##### Primipara

A mother who gave a live birth for the first time [19].

##### Colostrum avoidance

Colostrum avoidance includes: delayed initiation of breast-feeding; pumping and discarding colostrum; and/or wet nursing [3].

##### Pre-lacteal feeding

If an infant during the first 3 days of life took something other than breast milk [26].

##### Early initiation of breastfeeding

If an infant within 1 h of birth is put on the mother‘s breast to feed [27] .

##### Exclusive breastfeeding

Infant fed on only breast milk (with the exception ordered medicines and vitamins by health professionals 1 day (24 h.) before the survey was conducted [24].

##### Husband support

Husband who supports, encourages, and promotes the mother’s breastfeeding practice [28].

### Statistical analysis

The collected data were checked for completeness and consistency and then, coded and entered into EpiData 3.1 and exported to SPSS version 20 for analysis. Bivariate logistic regression was performed to each independent variable with the dependent variables. Then, variables with *p*-value < 0.25 were included in multivariate logistics regression analyses. The strength of association was measured using odds ratio and 95% confidence intervals. Statistical significance was declared at *P*-value ≤0.05.

### Ethics approval and consent to participate

Ethical approval was obtained from the research review ethical committee of the Addis Ababa University, and permission letter was obtained from Bahir Dar city mayor’s office. Data collectors informed each respondent about the study. Written and verbal consent was obtained from each study participants and confidentiality was assured for all information provided by not exposing to third body. Moreover, personal identifiers were not included in the questionnaire.

## Result

### Socio demographic profiles of participants

All participants were primipara mothers who were living in Bahir Dar city for 6 months prior the commencement of the study. From 400 eligible mothers, 398 were interviewed in this study making the response rate 99.5%. The mean age of mothers was 26 years with a standard deviation of (SD ± 4). More than half of the mothers (52%) were in the age range of 15–29 years. Out of 398, 73.9% were unemployed, 63.1% were uneducated, and 69.6% mothers belong to Orthodox Christianity while majorities (87.4%) belong to Amhara ethnic group. Majority of participants (86.7%) were married. Almost one out of ten had household income < 1500 Ethiopian Birr (Table 1).

**Table 1:** Socio-demographic Characteristics of first-time mothers having infants less than 6 months old, in Bahir Dar City, Northwest Ethiopia, 2016

Variable	Category(*n* = 398)	Frequency	Percent (%)
Sex of infant	Male	212	53.3
	Female	186	46.7
Age of infant (in months)	0–2.9 months	209	52.5
	3–3.9 months	99	24.9
	4–4.9 months	38	9.5
	5–5.9 months	52	13.1
Age of mother (in years)	15–29 years	207	52.0
	30–49 years	191	48.0
Religion of mother	Orthodox	277	69.6
	Muslims	95	23.9
	Others ^a*^	26	6.5
Ethnic of the Mother	Amhara	348	87.4
	Oromo	32	8.0
	Others ^b*^	18	4.5
Educational Level of mother	Uneducated	251	63.1
	Educated	147	36.9
Marital status of mother	Married	345	86.7
	Unmarried ^c*^	53	13.3
occupation of mother	Unemployed ^d *^	294	73.9
	Employed	104	26.1
Father’s Educational level(*n* = 344)	Uneducated	154	44.8
	Educated	190	55.2
Father’s occupation(*n* = 344)	Employed	184	53.5
	Unemployed	160	46.5
Type of family	Nuclear	323	81.2
	Extended	75	18.8
Household income ^e*^	< 1500 Birr	35	8.8
	> 1500 Birr	363	91.2

a* **=** Catholic, Protestant, Jehovah; b* = Tigrie, Agaw, Gurage; c* = single, divorced, widowed, d * = house wife, daily laborers, e* = 1 $US = 17 Birr

(Ethiopian)

### Health care service utilization profile of participants

Regarding to health care service utilization, nine out of ten participants got antenatal care (ANC) follow up. Amongst these, 60.9% of participants attended at health centers, 63.4% had four and more visits while 76.5% got breastfeeding counseling during ANC visits. Out of 398 participants, 86.4% gave birth at health institutions and majorities (82.2%) were delivered via normal delivery (Table 2).

**Table 2:** Maternal health service utilization and breastfeeding related factors first-time mothers having infants less than 6 months old, in Bahir Dar City, North west Ethiopia, 2016

Variable	Category (*n* = 398)	Frequency	Percent (%)
Got ANC follow up	Yes	361	90.7
	No	37	9.3
Place of ANC (*n* = 361)	Hospital	67	18.6
	Health Centre	220	60.9
	Private clinic	74	20.5
Number of ANC(*n* = 361)	< 3 times	132	36.6
	≥4times	229	63.4
ANC breastfeeding counseling (*n* = 361)	Yes	276	76.5
	No	85	23.5
Place of delivery	Health institutions	344	86.4
	Home	54	13.6
Mode of delivery	Normal/vaginal	327	82.2
	C/S	71	17.8
Early initiation of breast feeding	< 1 h	259	65.1
	> 1 h	139	34.9
Breast fed exclusively	Yes	228	57.3
	No	170	42.7
Colostrum avoidance	Yes	35	8.8
	No	363	91.2
Reasons to avoid colostrum	infant unable to feed	34	8.5
	Bad for infant	140	35.2
	yellow and creamiest	156	39.2
	Due tradition/culture	68	17.1
Pre-lacteal feeding practice	Yes	63	15.8
	No	335	84.2
Prelacteal foods given(*n* = 63)	Water	20	31.8
	Butter	30	47.6
	Others ^a*^	13	20.6
			17.1
Who influenced you to give other foods?	My Own Decision	90	24.2
	My Husband	108	29.0
	My Mother	76	20.4
	My Mother In Law	58	15.6
	Others ^b*^	40	10.8
Faced any breast feeding problem?	Yes	264	66.3
	No	134	33.7
Husband support in breast feeding(*n* = 397)	Yes	355	89.4
	No	42	10.6
Cultural support of breast feeding	Yes	52	13.1
	No	346	86.9

a* = cow’s milk, sugar solution, Honey b* = Friends, neighbors, members of extended family

### Colostrum avoidance and breast feeding practices

In the current study, out of 398 primipara mothers, 8.8% discarded colostrum. The most common reasons for discarding colostrum were; yellow and creamy (39.2%), bad for infant (35.2%), traditional/cultural reason (17.1%) and infant unable to feed (8.5%). Regarding breast feeding practices, 65.1% initiated breastfeeding within 1 h, 57.3% practiced exclusive breastfeeding to 6 months and 15.8% gave prelacteal feeding. Among those who gave prelacteal feeding, 47.6% gave butter, 31.8% gave water, and 20.6% gave other foods like cow milk and sugar solution (Table 2).

### Factors associated with colostrum avoidance and prelacteal feeding

In bivariate analysis, marital status, mother’s occupational status, mother’s educational status, father’s educational status, number ANC visits, mode of delivery, initiation of breastfeeding within 1 h and house hold income were statistically associated with colostrum avoidance. However, marital status, maternal occupation, mode of delivery and initiation of breastfeeding within 1 h were significantly associated with colostrum avoidance in multivariate analysis.

Marital status was significantly associated with colostrum avoidance. Married mothers were almost four times less likely to discard colostrum (AOR = 4.52, 95%CI: 1.13, 18.16) compared with unmarried mothers. Similarly, maternal occupation was significantly associated with colostrum avoidance. Mothers who were unemployed were about three times less likely to discard colostrum (AOR = 3.46, 95%CI: 1.15, 10.51) compared to mothers who were employed. Mode of delivery was also significantly associated with colostrum avoidance. Mothers who were underwent normal delivery were five times less likely to discard colostrum (AOR = 5.20, 95%CI: 1.87, 20.90) compared with mothers who were underwent caesarian delivery. Likewise, initiation of breastfeeding within 1 h was significantly associated with colostrum avoidance. Mothers who initiated breastfeeding within 1 h were almost 3 times less likely to discard colostrum (AOR = 2.79, 95%CI: 0.96, 8.16) compared with mothers who did not initiated breastfeeding within 1 h (Table 3).

**Table 3:** Factors associated with colostrum avoidance among primipara mothers having infants less than 6 months in Bahir Dar City, North west Ethiopia, 2016

	Colostrum Avoidance				
	Yes	No	COR (95%CI)	AOR (95%CI)	*P*-value
Variable	(N & %)	(N& %)			
Marital status					
Married	25 (7.2)	320 (92.8)	1		
Unmarried	10 (18.9)	43 (81.1)	2.98 (1.34,6.62)	4.52 (1.13,18.16)	0.033*
Maternal occupation					
Unemployed	21 (7.1)	273 (92.9)	1		
Employed	14 (13.5)	90 (86.5)	2.02 (0.987,4.4)	3.46 (1.15,10.51)	0.027*
Mother’s education					
Uneducated	29 (11.6)	222 (88.4)	3.07 (1.24,7.58)	0.28 (0.067,1.13)	.074
Educated	6 (4.1)	141 (95.9)	1		
Father’s education					
Uneducated	21 (13.6)	133 (86.4)	2.84 (1.30,6.24)	0.21 (0.07,.67)	0.09
Educated	10 (5.3)	180 (94.7)	1		
Number of ANC follow up visits					
< 3visits	12 (9.1)	120 (90.9)	1.81 (0.788,4.5)	0.93 (0.30,2.85)	0.894
≥ 4visits	12 (5.2)	217 (94.8)	1		
Mode of delivery					
Normal/vaginal	18 (5.5)	309 (94.5)	1		
C/S	17 (23.9)	54 (76.1)	5.4 (2.62,11.14)	5.20 (1.87,20.90)	0.003*
Early initiation of breast feeding					
Yes	14 (5.4)	245 (94.6)	1		
No	21 (15.1)	118 (84.9)	3.11 (1.53,6.34)	2.79 (0.96,8.16)	0.05*
Household income					
< 1500 birr	15 (42.9)	20 (57.1)	12.86 (5.74,28.8)	0.07 (0.02,0.35)	0.10
> 1500 birr	20 (5.5)	343 (94.5)			

*ANC* Antenatal Care, *C/S* Caesarian Section

*p-value ≤0.05 (Significant)

## Discussion

The feeding of infant has important implications for immediate and future health in developing countries like Ethiopia that have high rate of under-five malnutrition, morbidity and mortality. Colostrum is highly nutritious and immunogenic. However, its avoidance has been reported across the globe including Ethiopia. Its avoidance leads to the introduction of prelacteal feeding which is harmful practice for the newborn baby. The extent and associated factors of colostrum avoidance among premiparious mothers is not known in the study area. Identifying the extent and associated factors of colostrum avoidance has important implications to improve infant feeding practices and child health.

In this study, rate of colostrum avoidance and its associated factors were assessed. The current study revealed that colostrum was discarded by 8.8% of primipara mothers. The result is nearly in line with previous studies conducted in Ethiopia (8%) [14], Debretabor, Ethiopia (10.5%) [29], Kombolcha, Ethiopia (11.4%) [17], Tigray, Ethiopia (15%) [16] and Afar, Ethiopia (15.6%) [18]. The correspondence of the result might be due the similarity of study populations in culture, health care policy system, health care service utilization, methodology and socioeconomic factors.

However, the result is slightly higher than the results of the previous studies conducted in Indonesia (6.2%) [8] and Tigray, Ethiopia (6.3%) [11]. The dissimilarity of the results could be due socioeconomic differences, cultural differences and ethnic differences between populations. For example, Ethiopian and Indonesian people have different socioeconomic status, different cultural and ritual practices regarding child birth and newborn feeding. In addition, *Amhara* and *Tigray* are the two different ethnic groups found in Ethiopia having their own cultural and ritual practices related to child birth and newborn feeding practices.

On the other hand, the result of this study is far more lower than the results of previous studies conducted in Debre Birhan, Ethiopia (20%) [12], Bangladesh (37%) [30], rural northern Ethiopia (63%) [3], India (92%) [9], and (76.9%) [13] in Afar, Ethiopia. The variation in the rate of colostrum avoidance could be due to differences in cultural practices and beliefs, socioeconomic status, health care service utilization, demographic profile of study populations, time of studies conducted and methods used to conduct studies. For example, a study conducted in rural northern Ethiopia is a qualitative study while the current study is the quantitative one. Moreover, the previous study was conducted in the rural area while the current study was conducted in the urban area. From this explanation, it is clear that there is a methodological variation between the above mentioned studies.

In this study, colostrum avoidance was statistically associated with marital status, maternal occupation, and mode of delivery and timely initiation of breastfeeding.

Marital status was significantly associated with colostrum avoidance. Married mothers were almost 4 times less likely to discard colostrum compared with unmarried mothers. The finding is similar with the study from North Wolo, Ethiopia [31]. The possible explanation might be due to the fact that married mothers could have get social support from their husband during breast feeding practice. Previous studies have confirmed that husband support has a positive impact on breast feeding practices [24, 32]. In addition, a study conducted in United Kingdom to explore fathers’ experience of breast feeding promotion also revealed that mothers who have strong social support from their husband are more likely to practice early initiation of breastfeeding and colostrum feeding than who do not have support [28].

Maternal occupation was also found significantly associated with colostrum avoidance. Unemployed mothers were nearly 3 times less likely to discard colostrum. The finding is similar with the previous study done in Kombolcha, Ethiopia [17]. This could be likely due to unemployed could have more time to practice breastfeeding than employed mothers. Tewabe et al. have demonstrated in their work that unemployed mothers practiced breast feeding better than employed mothers [24].

Mode of delivery was also significantly associated with colostrum avoidance. Mothers who were underwent normal vaginal delivery were less likely to discard colostrum than mothers who were underwent cesarean section. The finding is in line with previous studies in Egypt and Vietnam [6, 33]. The possible explanation could be due to that mothers underwent cesarean section could have difficulties to initiate breast feeding due to post-surgical pain and complications. This in turn could delay early initiation of breast feeding and leads to colostrum avoidance. A study from turkey has demonstrated that mothers underwent cesarean section had high rate of delayed initiation of breast feeding [34]. Mukherjee et al. in their review of colostrum feeding practice worldwide also showed that delayed initiation of breastfeeding was observed among mothers underwent cesarean section [7]. This implies that strategies should be designed to prevent unnecessary cesarean section that negatively affects breast feeding practices particularly early initiation of breastfeeding and colostrum feeding. Health care workers who work in maternity units should give special attention to mothers who underwent cesarean section delivery. They should directly assist mothers to initiate breastfeeding and feed colostrum to their babies in addition to managing their post-surgical pain and discomforts.

Early initiation of breast feeding was significantly associated with colostrum avoidance. Mothers who initiated breastfeeding within 1 h were less likely to discard colostrum. The finding is in line with previous studies [15, 31]. The possible explanation might be due to the fact that if the mother delay to start breast feeding, she is likely to carry out infant feeding malpractices including colostrum avoidance. A study in Afar, Ethiopia done on Rural Pastoralist Community has revealed that delayed initiation of breast feeding leads to colostrum avoidance [13]. This implies that primigavida mothers should be counseled on early initiation of breast feeding during their antenatal care follow up. Mothers who missed antenatal care follow up should be informed about the importance of early initiation of breast feeding and feeding colostrum to their new born babies via public media like radio and television.

### Limitation

The study has some limitations. First, the study was cross-sectional so that the cause and effect relationship cannot be determined. Second, the study did not assess the qualitative aspect of colostrum avoidance. Third, husbands were not included in the study in spite of they have important role to play in deciding infant feeding practices. Finally, the information obtained from mothers could be subjected to recall bias.

## Conclusion

This study revealed that colostrum avoidance practice is common among primipara mothers in the study area. Marital status, maternal occupation, and mode of delivery and timely initiation of breastfeeding were predictors of colostrum avoidance in primipara mothers. Health care workers, who work in maternity unity should give attention to unmarried and employed mothers, should discourage unnecessary cesarean section deliveries and promote early initiation of breast feeding to improve colostrum feeding practice. As this study is the first study done on primipara mothers in the country, it could be used as a base line study for the future researchers. Finally, further interventional and longitudinal studies are needed to improve colostrum feeding practice among primipara mothers.

## Data Availability

The data of this study can’t be shared publically due to the presence of sensitive (confidential) participants’ information. The data can be obtained from corresponding author up on reasonable request.

## Abbreviations

ANC: Antenatal Care
C/S: Caesarian Section
AOR: Adjusted Odds Ratio
CL: Confidence Level
COR: Crude Odds Ratio
AOR: Adjusted Odds Ratio
EDHS: Ethiopian Demographic Health Survey
SD: Standard Deviation
WHO: World Health Organization
UNICEF: United Nation Children’s Fund
SPSS: Statistical Package for the Social Sciences
HIS: Health Information System
